# Consumer Description by Check-All-That-Apply Questions (CATA) of the Sensory Profiles of Commercial and New Mandarins. Identification of Preference Patterns and Drivers of Liking

**DOI:** 10.3390/foods9040468

**Published:** 2020-04-09

**Authors:** Paula Tarancón, Amparo Tárrega, Pablo Aleza, Cristina Besada

**Affiliations:** 1 Centro de Tecnología Postcosecha, Valencian Institute for Agricultural Research (IVIA), Carretera Moncada-Náquera, km. 4.5, 46113 Moncada, Spain; tarancon_pau@gva.es; 2 Instituto de Agroquímica y Tecnología de Alimentos (IATA-CSIC), Agustín Escardino 7, 46980 Paterna, Spain; atarrega@iata.csic.es; 3 Centro de Citricultura y Producción Vegetal, Valencian Institute for Agricultural Research (IVIA), Carretera Moncada-Náquera, km. 4.5, 46113 Moncada, Spain; aleza@ivia.es

**Keywords:** taste, aroma, sensory attributes, physico-chemical properties

## Abstract

In the last few years, the interest in developing new mandarin cultivars of superior quality has grown as a response to the increasing consumer demand of this appreciated fruit. This study evaluated the sensory profiles of five new late-season mandarin cultivars (‘Alborea‘, ’Coral‘, ’Omet‘, ’Matiz‘ and ’Tri-703‘) and six commercial cultivars found contemporarily in stores (‘Clemenules‘, ’Nova‘, ’Tango‘, ’Nadorcott‘, ‘Orri’ and ‘Ortanique’). The sensory profiles of the cultivars were described by consumers through CATA questions. Consumers’ acceptability and the main physico-chemical properties were also evaluated. Twenty-two out of 23 CATA terms differed significantly for the sensory profiles of the studied cultivars. The new cultivars shared a similar profile, described mainly as “very intense taste”, “refreshing taste”, “very aromatic” and “juicy”, and these characteristics were quite different from those of the commercial cultivars. By combining acceptability and CATA questions, drivers of liking for segments of consumers with different preference patterns were identified. This is the first time that the sensory profiles of mandarin cultivars have been described by consumers. A significant number of consumers preferred the new mandarin cultivars to the commercial ones, which allows them a promising introduction on the market.

## 1. Introduction

Among citrus species, the consumption of mandarins has significantly increased in the last decade [1]. Mandarin fruits have a delicate appealing flavor that derives from a blend of sweet, sour, fruity and fresh characteristics [2].

As in other fruits, in citrus fruit, it is crucial to know the relation between sensory properties and instrumental measurements of quality. In this sense, sweet and acid are key attributes for the taste of citrus fruit and, therefore, for their overall quality. The Maturity Index (MI = total soluble solids/acidity) is normally used to express the relation between these two parameters as a unique value. In fact, the internal quality requirements for exporting and marketing of citrus fruit in the European Union are based on a minimum MI value and juice yield (JY). The minimum criteria required at harvest for commercializing mandarins depends on the group to which the cultivar belongs (Satsumas: MI > 6.5 and JY > 33%; Clementines: MI > 7 and JY > 40%; other cultivars and their hybrids: MI > 7.5 and JY > 33%) [3].

Apart from their flavor and taste, mandarins have particular traits related to convenient consumption, such as seedlessness, easiness to peel or segment separation, which also contribute to their global quality and become more important in recent years for consumer satisfaction. Recently, rind color has been also shown to be a decisive factor for purchase intention [4].

In this context, the Valencian Institute of Agriculture Research (IVIA), located in the most important mandarin-growing area in Spain, hosts a breeding program to obtain new seedless late-season mandarins of high sensory quality [5]. Currently, the IVIA breeding program has several mandarin hybrids that have been preselected based on their late harvest date and good agronomic behavior. In addition to these selection criteria, to introduce new cultivars with a certain guarantee of success, it is necessary to study consumer acceptance to predict market response. Consumer tests are a valid tool to fulfill this goal, as shown by several studies, in which promising cultivars of kiwis, pears or cherry tomatoes have been identified based on consumer responses [6,7,8].

Furthermore, if consumers’ acceptance tests are combined with the description of fruit sensory attributes, very valuable information is obtained, as drivers of liking can be identified. This would be useful information for the citrus sector as it would help not only growers and marketers to make production and marketing decisions, but also breeders to develop new cultivars [9]. This kind of studies has been traditionally conducted by combining descriptive analyses, carried out by an expert or trained panel, with the evaluation of consumers’ acceptance [10]. The first test provides data on the sensory attributes of samples and the latter test indicates how much consumers like samples [11]. This methodology has been used to identify key drivers of liking in fruits such as mangos, strawberries, nectarines and peaches [12,13,14]. Regarding mandarins, Goldenberg et al. [1] identified the key factors influencing mandarin flavor liking after studying a group of cultivars from an Israeli citrus breeding collection.

In the last decade, the use of novel methodologies, such as Check All That Apply (CATA), has become a valuable alternative tool to the traditional descriptive analysis to characterize food sensory properties. In this method, consumers are provided with a list of words or phrases from which they should select all the words that they consider appropriate to describe a product. This methodology is less time-consuming and more flexible than traditional methods, and it allows for a more realistic approach to consumer perceptions [15,16,17,18,19]. CATA was successfully used to describe the sensory attributes of new cultivars of fruits such as strawberry [20,21]. These studies proved that CATA questions were able to provide information about consumer perceptions of the sensory properties, which could be very useful when selecting cultivars while evaluating new genotypes.

To select new cultivars based on their success chances when released to markets, sensory studies should take into account the commercial context; that is, they should cover not only new cultivars, but also the commercial ones present contemporarily in stores [22,23]. Any newly released cultivar that is intended to replace or compete with the cultivars already available on the market must be comparable or of superior quality for consumers [24].

In this context, this work aimed to study consumer responses to five new late season cultivars recently obtained at the IVIA in comparison to commercial cultivars contemporarily found on the market. To this end, consumer sensory perceptions of the different cultivars were studied by assessing their acceptance and describing sensory properties using the CATA method. This allowed us to identify the sensory characteristics that could be considered drivers of liking for mandarins as well as the most promising cultivars among the new ones.

## 2. Materials and Methods

### 2.1. Samples

Eleven mandarin cultivars were considered in the present study. The eleven samples comprised of five late season and seedless cultivars developed by the IVIA: ‘Alborea’, ‘Coral’, ‘Omet’, ‘Matiz’ and ‘Tri-703’. As the new considered cultivars had different harvesting periods, the study implied two harvest dates. ‘Alborea’ and ‘Coral’ were harvested at the end of January (Harvest 1) and ‘Omet’, ‘Matiz’ and ‘Tri-703’ at the end of February (Harvest 2). The new cultivars were assessed by comparing them to the commercial cultivars chosen according to the market availability of each harvesting period. Thus ‘Alborea’ and ‘Coral’ were compared to ‘Clemenules’, ‘Nova’ and ‘Tango’ and ‘Omet’, ‘Matiz’ and ‘Tri-703’ to ‘Nadorcott’, ‘Orri’ and ‘Ortanique’.

The new cultivars under study were harvested at their commercial maturity stage from the IVIA’s experimental orchards. The commercial ones were obtained upon their arrival at a commercial packing house located in Valencia (the FONTESTAD S.A. Company, Spain). All the commercial cultivars were harvested from orchards located in Valencia and were collected within one day following harvest. Fruit were taken to the Postharvest Department of the IVIA where they were selected according to homogenous color and absence of external defects.

### 2.2. Physico-Chemical Evaluation

Peel color was measured by a Minolta colorimeter (model CR-300; Minolta Co. Ltd., Osaka, Japan) using 20 fruits per cultivar and two measurements were taken in the equatorial zone of each fruit. The mean lightness (L), red-green (a) and yellow-blue (b) Hunter parameter values were calculated for each fruit and expressed as a color index (CI = 1000 a/Lb) [25].

Firmness measurements were taken by an Instron Universal Testing Machine (model 3343, Instron Ltd., Buckinghamshire, UK) on 20 fruits per treatment. The results were expressed as the percentage of millimeters of fruit deformation that resulted from 10 N pressure, applied by a 3.5 cm plunger on the longitudinal axis at constant speed.

For the total soluble solids contents (TSS) and titratable acidity (TA), four samples of five fruits each per treatment were squeezed in an electric juice extractor with a rotating head (Lomi^®^, Model 4, Lorenzo Miguel, S.L., Madrid, Spain). Firstly, the juice yield was measured and expressed as a percentage, calculated by dividing the volume of juice by the total fruit weight. Then, TA was determined by titration with 0.1 N NaOH solution, using phenolphthalein as the indicator, and was expressed as grams of citric acid per 100 mL of juice. The TSS in juice was determined by a digital refractometer (Atago PR-1, Atago Co., Ltd., Tokyo, Japan) and data were expressed as percent. The ripening level was expressed according to the maturity index calculated as TSS/TA.

### 2.3. Consumer Study

In total, 128 consumers participated in the test corresponding to Harvest 1 and 139 in that corresponding to Harvest 2. For both tests, cultivars were assessed by evaluating acceptance and the CATA questions. Consumers were recruited from the IVIA, the Universidad Politécnica de Valencia (UPV) and the Instituto de Agroquímica y Tecnología de Alimentos (IATA-CSIC), and were selected according to their interest in participating. The whole group of participants was made up with administration staff and students; hence, they were not linked to food research field and can be considered naïve consumers. They were aged between 18 and 65, and the male/female ratio (%) was 43/57 and 46/54 for Harvests 1 and 2, respectively.

The evaluations were carried out in a standardized test room (ISO 8589; ISO 2007) [26]. Mandarins were served whole in cups coded with three-digit random numbers and were presented monadically following a Williams’ Latin square design [27], and consumers were provided with water to cleanse palate between samples. The consumers were instructed to first peel the mandarin, then taste it and score their overall acceptance on a nine-point hedonic scale ranging from 1 (“dislike extremely”) to 9 (“like extremely”). Afterwards, they were asked to answer the Check All That Apply questions, comprising of 23 descriptors. The descriptors were related to the peeling characteristics, flavor, taste and texture of mandarins. The descriptors were initially selected based on previous research [1,2] and were adapted according to the specific characteristics of samples. To this end, a group of eight semi-trained panelists (people used to generate attributes) evaluated the samples in order to add attributes to the list. Finally, a group of eight consumers participated in a session to generate the final list of attributes that would be included in the CATA questions. They were given the samples, a list of the potential attributes and an outline of the procedure. The assessors were asked to taste the samples, and write down the most appropriate attributes to describe each one; they could use the terms in the list, but they were encouraged to suggest new terms that were relevant for describing the mandarins they tasted. All this information was used to gather a final list of attributes, in which, based on consumers descriptions, different intensities were included for some of the attributes. This design was previously described by Lado et al. [20], who reported that CATA questions may provide information about consumers’ perception of the intensity of sensory attributes of a food product, particularly fruit cultivars. In the final list, the attributes were ordered as they are likely to be perceived. The selected descriptors were: intense odor when starting to peel, hard starting to peeling, easy to peel, difficult to peel, stain hands when peeling, tasteless/dull, not very sweet, sweet, very sweet, overripe taste, not traditional/novel taste, refreshing taste, traditional mandarin taste, not very sour, sour, very sour, not very aromatic, very aromatic, very intense taste, soft, fibrous, juicy and juiceless.

Finally, when consumers had finished the mandarin’s assessment, they answered a few demographic questions such as gender, age and frequency of mandarin/orange consumption during the season. The response options for fruit consumption were as follows: “twice a week or more”, “once a week” or “2 or 3 times a month”.

### 2.4. Statistical Analysis

A one-way analysis of variance (ANOVA) was used to evaluate the difference in the physico-chemical parameters among cultivars. In this analysis the treatments were the cultivars and significant difference between them were determined by calculating the Least Significant Difference test (*p* ≤ 0.05). The Kruskall–Wallis test followed by Dunn´s multiple comparisons test were applied to evaluate difference in acceptance scores among cultivars (*p* ≤ 0.05).

To assess the dispersion of the acceptability data, descriptive statistics were calculated and expressed as box-and-whisker plots. A hierarchical cluster analysis (HCA) was performed with the liking data to identify groups of consumers with similar preference patterns. Euclidean distances (dissimilarity), Ward’s techniques (agglomeration method) and automatic truncation were used.

The frequencies of citation of attributes in CATA were determined for each sample. The non-parametric Cochran’s Q test was performed on the raw binary CATA data to determine significance among samples for each sensory attribute (*p* ≤ 0.05).

A Multiple Factor Analysis (MFA) was performed with the dataset of each harvest to investigate the relationship between responses to the CATA questions of the three groups of consumers identified in the cluster analysis. For Harvest 1, this involved the construction of a five-row matrix (cultivars) with three groups of columns, which corresponded to the terms used by the consumers for describing the samples in each cluster. The same was done for Harvest 2, but with a six-row matrix. Moreover, in both cases, liking data were projected as supplementary variables.

In addition, penalty analyses of the CATA [28] data were performed for global liking, to determine the drop in global liking associated with a deviation for each attribute.

Finally, MFA was performed to study the relation between the physico-chemical and sensory properties of all the studied cultivars.

All the calculations were carried out with the XLSTAT software (version 2019, Addinsoft Inc. New York, NY, USA).

## 3. Results and Discussion

### 3.1. Physico-Chemical Parameters

According to results shown in Table 1, in which the cultivars harvested at the same time were compared to each other, the commercial cultivars ‘Clemenules’ and ‘Nova’ had the highest MI at Harvest 1, with MI values of 27.6 and 16.8, respectively (Table 1). The other cultivars had a similar MI to one another, with values between 10.8 and 13.1. As previously explained, this index is related to the TSS and the acidity parameters. ‘Alborea’ had the highest TSS among all the studied cultivars (13.4%) while the other cultivars obtained values around 12% with no significant difference. Thus, the difference in MI among cultivars were due mainly to the acidity level, which was significantly higher in ‘Tango’, ‘Alborea’ and ‘Coral’ (with values slightly higher than 1 g citric ac./100 mL) compared to cultivars ‘Clemenules’ and ‘Nova’ (0.7 and 0.4 g citric ac./100 mL, respectively). It is important to emphasize that the harvest window of the studied cultivars is not the same. That is, when Harvest 1 was carried out, at the end of January, commercial cultivars ‘Clemenules’ and ‘Nova’ were at the end of their harvest period, while ‘Tango’ and the new cultivars ‘Alborea’ and ‘Coral’ were in the early-mid stages of their season. This explains the higher MI of ‘Clemenules’ and ‘Nova’ as the fruits from these two cultivars had reached a more advanced maturity stage. In this study, the heterogeneity of MI at harvest clearly reflects what actually happens as the harvest window of the different cultivars may not be the same, but they often overlap to a greater or lesser extent. Therefore, it is common to find fruit contemporarily on the market in different maturity stages.

Regarding the Harvest 2 cultivars, the lowest MI was detected in ‘Ortanique’ (MI = 7.9), while the highest value was for ‘Orri’ (MI = 19.5). The other cultivars showed values between 9.2 and 11.6, with slight difference among them. Once again, the main difference in MI were due to difference in acidity levels. The commercial cultivar ‘Orri’ had the lowest acidity level, while the most acidic cultivars were ‘Matiz’, ‘Omet’ and ‘Ortanique’. TSS ranged between 11.6% and 14.6%, and the new cultivars ‘Tri-703’ and ‘Omet’ showed the highest TSS content, while the lowest content was detected in ‘Nadorcott’ and ‘Ortanique’. In this case, all six cultivars can be considered as in the early-mid stages of their season.

As ‘Clemenules’ belongs to the Clementines group and all the other cultivars in this study are classified in the “other varieties and their hybrids” group, we affirm that the MI requirements for marketing, which are detailed in the Introduction [3], were met for all the cultivars herein studied. Moreover, they all showed juice yield values between 45% and 58%, which were, therefore, much higher than the minimum required values.

Regarding texture, of the cultivars assessed in Harvest 1, the lowest firmness values were detected for commercial cultivars ‘Tango’ and ‘Clemenules’ (>6% deformation), while the other cultivars gave deformation values of around 4%. In general, the cultivars from Harvest 2 showed higher firmness than those from Harvest 1. ‘Omet’ was the firmest cultivar (2.1% deformation), ‘Nadorcott’ was the softest (5.4% deformation) and the rest showed deformation values between 2.6% and 4.3%.

Regarding the weight of cultivars under study, for Harvest 1, the variety with the lowest significant weight was ‘Tango’ (about 82 g per fruit). Among the other cultivars, no significant differences in weight were found, which ranged from 95 to 110 g per fruit. The mean weight for the Harvest 2 cultivars ranged from 98.6 to 180.5 g per fruit, and the weight of ‘Ortanique’ was significantly heavier compared to the other cultivars.

### 3.2. Consumer Responses

#### 3.2.1. Identification of Preference Patterns

**Figure 1:**
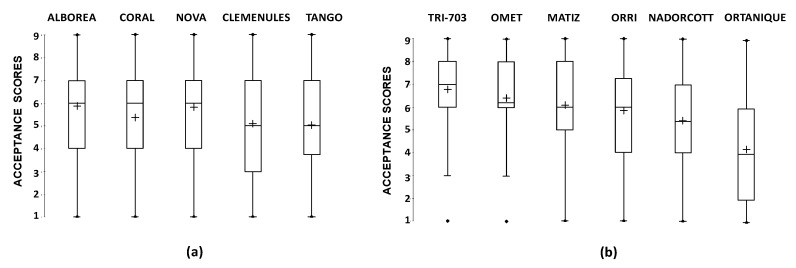
Box-and-wisher plots of descriptive statistics for consumers’ liking scores of the mandarin cultivars evaluated at: Harvest 1 (**a**); and Harvest 2 (**b**). The lower and upper edges of a box represent the first (Q1) and third (Q3) quartile, while the line and cross within the box show the median and the mean, respectively. The whiskers represent the lower and upper extremes of the data.

The mean acceptance scores ranged between 5.0 and 5.9 for the cultivars assessed in Harvest 1, and between 4.2 and 6.8 for those evaluated in Harvest 2. Box and whisker plots for each mandarin cultivar (Figure 1) revealed that the scores for most cultivars were spread over the whole scale range. Moreover, the difference between the first quartile (Q1, lower edge of the box) and third quartile (Q3, upper edge of the box) was between 3 and 4 points on the scale for most cultivars. Given the wide variability among consumers’ scores, as revealed by the box-and-whisker plots, a hierarchical cluster analysis was carried out separately for Harvests 1 and 2 to investigate in-depth consumer preferences for the different mandarin cultivars (Figure S1). In both cases, three different preference patterns were identified among all the consumers who participated in each test (Figure S1).

**Figure 2:**
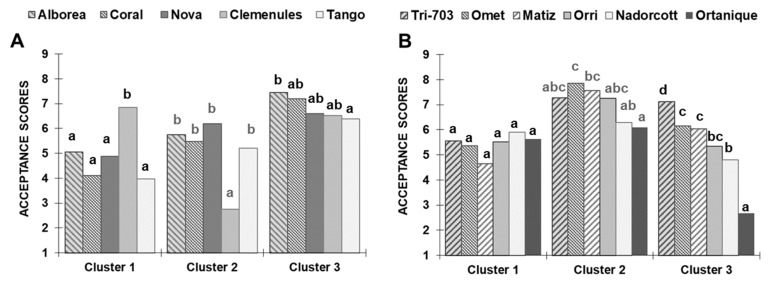
Mean acceptance scores of consumers (Clusters 1–3) who evaluated: Harvest 1 cultivars (**A**); and Harvest 2 cultivars (**B**). For each harvest, scores not sharing letters within each cluster were significantly different (*p* ≤ 0.05) according to Kruskal–Wallis test (two-tailed).

Among the three groups of consumers identified in the test corresponding to Harvest 1 (Figure 2a), Cluster 1 was made up of 46 consumers (36%), Cluster 2 had 53 consumers (41%) and Cluster 3 comprised of 29 consumers (23%). Significant difference in acceptance scores among cultivars were detected in the three clusters (Cluster 1: *p* < 0.0001, *K* = 56.4; Cluster 2: *p* < 0.0001, *K* = 75.01; Cluster 3: *p* = 0.025, *K* = 11.18). Our results show that consumers of Cluster 1 showed a marked preference for ‘Clemenules’ (mean scores of 6.8), while the mean scores of the other cultivars ranged between 5.0 and 3.9, with no difference among them. Contrarily, the consumers of Cluster 2 clearly disliked ‘Clemenules’ as this cultivar obtained the lowest score compared to the other cultivars, which obtained similar scores to one another. The consumers from Cluster 3 rated all the cultivars with higher scores than those of Clusters 1 and 2. For all the cultivars, scores ranged between 7.4 and 6.4, ‘Alborea’ being the cultivar that obtained the highest score.

Different preference patterns among segments of consumers were also described after evaluation of California mandarin and tangelo cultivars [29]. In the mentioned study, similar to that reported herein for ‘Clemenules’, the tangelo cultivar ‘Minneola’ was the most liked by a segment of consumers while other segments of people disliked it the most.

Regarding Harvest 2 (Figure 2b), Cluster 1 was made up of 33 consumers (23%), Cluster 2 had 35 consumers (25%) and Cluster 3 comprised of 71 consumers (51%). On the one hand, the consumers from Cluster 1 showed no preference for any studied cultivars as they all obtained scores of around 5–6, with no significant difference among them (*p* = 0.25, *K* = 6.54). On the other hand, significant difference in acceptance scores were detected in Clusters 2 and 3 (Cluster 2: *p* < 0.0001, *K* = 28.73; Cluster 3: *p* < 0.0001, *K* = 156.8). Although the consumers of these two clusters had different preference patterns according to the HCA, there were some similarities among them that are worth mentioning. In both cases, consumers gave the highest scores to the three new cultivars followed by ‘Orri’, ‘Nadorcott’ and ‘Ortanique’, but the consumers of Cluster 2 rated all the cultivars with higher liking scores than those in Cluster 3. Moreover, no differences were detected among the three new cultivars in Cluster 2 while ‘Tri 703’ was the most liked cultivar for the consumers of Cluster 3.

#### 3.2.2. CATA Questions

Tables S1 and S2 shows the frequency of mentioning all the terms used by consumers to describe the mandarins under study. In general, for both harvests, the most frequently used terms were: intense odor when starting to peel, easy to peel, difficult to peel, sweet, sour, very aromatic and juicy. According to the non-parametric Cochran’s Q test, significant differences were found in the frequencies of mentioning all the descriptors of the CATA questions presented to the consumers in Harvest 1 (Table S1). In Harvest 2, only the descriptor “fibrous” was non-significant and was removed from the subsequent statistical analyses (Table S2). These results corroborate that the CATA is a powerful tool to evaluate consumer perceptions of difference in the sensory attributes among the studied mandarin cultivars.

As in both harvests different groups of consumers were identified based on their preference patterns (Figure 2), MFA and penalty analysis were carried out by considering the CATA counts for the three consumer groups identified in each Harvest and the liking of each group.

##### Harvest 1

**Figure 3:**
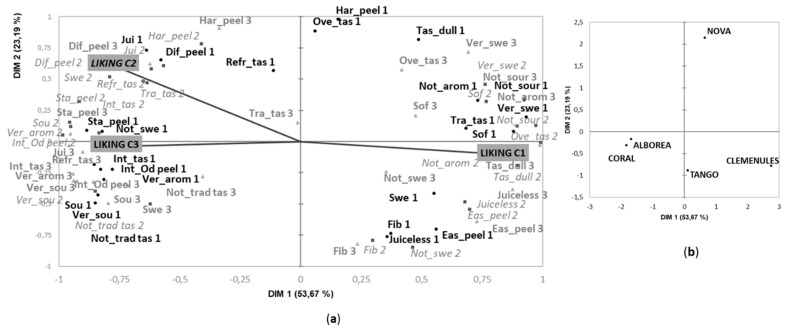
MFA of the CATA question in Harvest 1 considering responses for each of the three identified clusters as active datasets and mean liking scores as supplementary data: (**a**) vocabulary and liking (Cluster 1: black circle; Cluster 2: grey square; Cluster 3: light grey triangle); and (**b**) samples representation. DIM—dimension. Short forms of the CATA terms followed by numbers 1, 2 and 3 (corresponding to each cluster) appear in the MFA and their meanings are the following: Difficult to peel, *Dif_peel*; Easy to peel, *Eas_peel;* Intense odor when starting to peel, *Int_Od peel*; Stain hands when peeling, *Sta_peel*; Hard starting peeling, *Har_peel*; Very aromatic, *Ver_arom*; Not very aromatic, *Not_arom*; Refreshing taste, *Refr_tas*; Not traditional/novel taste, *Not_trad tas*; Not very sweet, *Not_swe*; Sweet, *Swe*; Very swee, *Ver_swe*; Tasteless/dull, *Tas_dull*; Very intense taste, *Int_tas*; Traditional mandarin taste, *Tra_tas*; Not very sour, *Not_sour*; Sour, *Sou*; Very sour, *Ver_sou*; Juicy, *Jui*; Soft, *Sof*; Overripe taste, *Ove_tas*; Juiceless, *Juiceless.*

The first two dimensions of the MFA for Harvest 1 explained 77% of variance (Figure 3). Cultivars ‘Coral’ and ‘Alborea’ were grouped together and were allocated in the left part of the first dimension while ‘Clemenules’ was allocated opposite along this dimension (Figure 3b). The other two cultivars, ‘Tango’ and ‘Nova’ were mainly differentiated for the second dimension. According to Figure 3a, consumers from Clusters 2 and 3 used the CATA terms in a similar way. Thus, the new cultivars ‘Coral’ and ‘Alborea’ were mainly described with the attributes very aromatic, intense odor when starting to peel, sweet, sour, very sour, very intense taste, refreshing taste and juicy. Moreover, based on results of penalty analysis (Figure S2) and the correlation of these attributes with the liking of C2 and C3 consumers in the MFA, these characteristics were identified as drivers of liking for these two groups of consumers. On the contrary, attributes such as tasteless/dull, not very sour, not very sweet or not very aromatic were disliked by these consumers (Figure S2), and they were mainly used to described ’Clemenules’ (Figure 3).

Interestingly, consumers of Cluster 1 used other attributes such as traditional mandarin taste, sweet and very sweet to describe ’Clemenules’, which was the variety that they liked the most. In fact, penalty analysis revealed “traditional mandarin taste” as the main driver of liking for this segment of consumers. They penalized characteristics such as not very sweet and tasteless/dull (Figure S2) that they used to describe the new cultivars ‘Coral’ and ‘Alborea’ and the commercial cultivar ‘Nova’, respectively (Figure 3).

Interestingly, when we compared the drivers of liking/disliking from the consumers of Clusters 2 and 3 to those of Cluster 1 (Figure S2), we observed that the terms related to taste, such as traditional mandarin taste or sour and sweetness perceptions, were crucial for liking in both consumer groups. However, the aroma and textural properties of mandarin (aroma intensity and juiciness) seemed important to a greater extent for the consumers of Clusters 2 and 3.

Regarding ‘Nova’ and ‘Tango’ cultivars, they were located in the middle area of the MFA, separated from the rest of the cultivars; this indicates that these two cultivars share some characteristic with ‘Clemenules’ but also other ones with ‘Alborea’ and ‘Coral’. Moreover, ‘Tango’ was characterized by all consumers as easy to peel and as being fibrous. In the case of ‘Nova’, consumers found it difficult to peel and, as previously mentioned, consumers of Cluster 1 considered it to have an overripe taste.

##### Harvest 2

**Figure 4:**
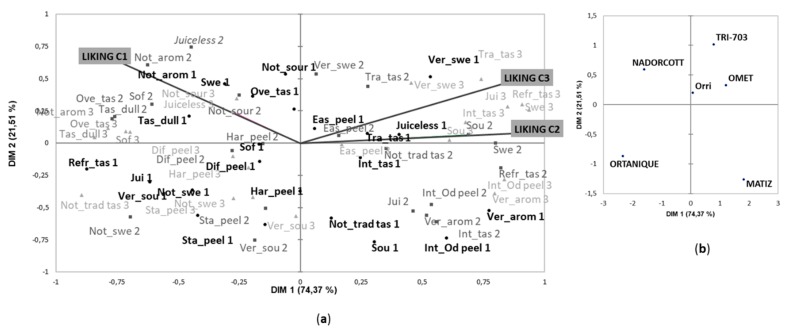
MFA of the CATA question in Harvest 2 considering responses for each of the three identified clusters as active datasets and mean liking scores as supplementary data: (**a**) vocabulary and liking (Cluster 1: black circle; Cluster 2: grey square; Cluster 3: light grey triangle); and (**b**) samples representation. DIM—dimension. Short forms of the CATA terms followed by numbers 1, 2 and 3 (corresponding to each cluster) appear in the MFA and their meanings are the following: Difficult to peel, *Dif_peel*; Easy to peel, *Eas_peel;* Intense odor when starting to peel, *Int_Od peel*; Stain hands when peeling, *Sta_peel*; Hard starting peeling, *Har_peel*; Very aromatic, *Ver_arom*; Not very aromatic, *Not_arom*; Refreshing taste, *Refr_tas*; Not traditional/novel taste, *Not_trad tas*; Not very sweet, *Not_swe*; Sweet, *Swe*; Very swee, *Ver_swe*; Tasteless/dull, *Tas_dull*; Very intense taste, *Int_tas*; Traditional mandarin taste, *Tra_tas*; Not very sour, *Not_sour*; Sour, *Sou*; Very sour, *Ver_sou*; Juicy, *Jui*; Soft, *Sof*; Overripe taste, *Ove_tas*; Juiceless, *Juiceless.*

In Harvest 2, the CATA terms were used similarly by the consumers of the three clusters and allowed for difference to be detected in the sensory characteristics of the mandarin cultivars. The first MFA dimension (74% of variance) contributed to clearly separate the new cultivars ‘Omet’, ‘Matiz’ and ‘Tri-703’ from ‘Nadorcott’ and ‘Ortanique’ and, to a lesser extent, from ‘Orri’ (Figure 4). Along the second dimension, which explained 21% of variance, ‘Ortanique’ and ‘Matiz’ were separated from all the other cultivars.

The three new cultivars were described as having a traditional and very intense taste, intense odor when starting to peel and as being juicy, sour, very aromatic and very sweet (Figure 4). Based on penalty analysis, these terms were identified as drivers of liking for the consumers of Clusters 2 and 3, who liked the new cultivars the most (Figure S3). Of the three new cultivars, ‘Matiz’ was highlighted by the intensity of its odor during peeling and eating. Of the commercial cultivars, ‘Ortanique’ was characterized mainly by its difficulty to peel, and it was also considered to be very acid and not very sweet. ‘Nadorcott’ was described as having less intense aroma and taste than the other cultivars and as being neither very sour nor juicy (Figure 4). Consumers of Cluster 1 described these two cultivars as having a refreshing taste, which positively affected their liking scores (Figure S3). ‘Orri’ cultivar was situated in the middle of the MFA space, which indicates that consumers were not able to clearly differentiate it from the other cultivars.

Even though not much research has been conducted into the sensory characterization and consumer responses to mandarins, Goldenberg et al. [1] reported that mandarins’ likeability was linked to the following attributes: high sweetness, moderate to low acidity levels, low bitterness and gumminess, strong fruity and mandarin flavor and high juiciness. Some of these attributes fall in line with the drivers of liking herein identified, but others such as refreshing taste, very aromatic and intense odor when starting to peel are reported for the first time. Our approach also allowed drivers of disliking to be identified. Contrarily to that reported in California [30], where a segment of consumers showed significant negative correlations with perceived sourness after evaluating 15 mandarin cultivars, in the present study, high levels of sourness were not identified as drivers of disliking (Figures S2 and S3). On the contrary, “not very sour” attribute was penalized by consumers of Cluster 2 and 3 in Harvest 1.

In this study, CATA questions included attributes with different intensities, which is not a common practice in this type of test. However, in this case, during the attributes generation step, consumers reported adverbs of quantity such as “very” and “not very” and the need of using them for better describing sensory differences among cultivars. This modality in CATA questions was previously used by Ares et al. [31] and Lado et al. [20] for the evaluation of chocolate milk desserts and strawberry cultivars, respectively. Consumers description of a food product by means of attributes with different intensities has been also reported after using the open-ended question test [32].

In agreement with Lado et al. [20], our results show that attributes preceded by the adverb “very” were negatively correlated with attributes with the quantitative expression “not very”, suggesting that the use of these terms by consumers was related to the intensity (Figure 3). Interestingly, our results also show that in the case of those attributes such as “sweet” and “sour” in which three different intensities were included in the CATA questions (not very sweet, sweet and very sweet and not very sour, sour and very sour), consumers used the different intensities to describe different cultivars. This was clearly observed in the Harvest 2 (Figure 4), when consumers of Cluster 1 used the attribute “not very sweet” to describe the cultivar ‘Ortanique’, the term “sweet” was majorly applied to ‘Nadorcott’ and “very sweet” was mainly used to describe the new cultivars ‘Tri-703’ and ‘Omet’. In the same way, consumers of Clusters 2 and 3 used “not very sour” for the description of ‘Nadorcott’, “sour” for the cultivars ‘Tri-703’ and ‘Omet’ and “very sour” for ‘Ortanique’.

Based on these results, including quantitative adverbs in the attributes list of a CATA test allows for a better description and differentiation among products in the case of attributes whose intensity is relevant for consumers (e.g., sweet and sour). However, it is important to keep in mind that the decision of including attributes with different intensities must be based on the preliminary consumers’ description of the samples under study.

### 3.3. Relationship between the Sensory and Instrumental Data

**Figure 5:**
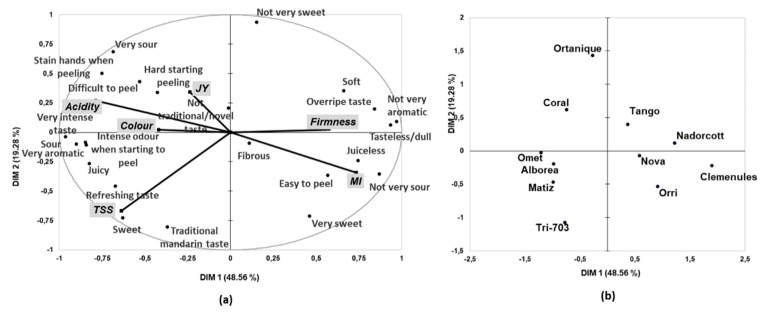
MFA using CATA counts and physico-chemical data of the eleven cultivars under study. (**a**) representation of the sensory attributes and physico-chemical properties on the first two dimensions of the MFA, (**b**) representation of the eleven mandarin cultivars on the first two dimensions of the MFA. DIM—dimension

To investigate correlations between sensory characteristics and instrumental variables, an MFA was carried out in the CATA counts for the 11 studied cultivars. As shown in Figure 5, the first two MFA dimensions accounted for 69% of the variance of the dataset.

Clearly, the first dimension separated attributes that were the opposite to one another (Figure 5a). Thus, terms such as soft, overripe taste, tasteless/dull, not very aromatic, juiceless, not very sour and very sweet were allocated in the right part of the MFA space, while terms such as very intense taste, sour, very aromatic, intense odor when starting to peel, juicy and refreshing taste were allocated in the left of the space.

On the one hand, most of the attributes that showed positive values on the first MFA dimension can be related to fruit in an advanced maturity stage (mainly overripe taste, soft, not very sour and very sweet). In fact, the MI correlated positively with these parameters, which corroborated this relation. Instrumental firmness also correlated positively with these attributes. It is noteworthy that fruit firmness was expressed as the percentage of deformation in fruit when a force of 10 N was applied. Therefore, the higher is the deformation percent, the softer is the fruit.

On the other hand, acidity was the main instrumental measurement to be positively linked to the attributes with negative values on the first dimension, such as sour, very sour, very intense taste, very aromatic or refreshing taste. In addition, peeling properties, such as difficult to peel, hard to starting peeling or stain hands when peeling, were also positively linked to instrumental acidity and negatively correlated with the MI. Finally, it was interesting to observe that the instrumental measurement of TSS, situated in the lower left part of the MFA space, correlated positively with not only sweetness perception, but also with traditional mandarin taste.

Therefore, our results show that TSS content, acid content, the MI and firmness measurements correlated quite well with consumer perceptions of the intensity of sensory attributes such as sweetness, sourness, textural and peeling properties. Thus, the MFA results demonstrate that consumer descriptions of mandarins by means of the CATA questions were in accordance with the physico-chemical characteristics of fruit.

Figure 5b shows the distribution of cultivars on the first two dimensions of the MFA. By combining the cultivars in Harvests 1 and 2, we gained an overview of the similarities/difference among all the variables in this study. Interestingly, the five new cultivars were all allocated on the left of the MFA space, with ‘Omet’, ‘Matiz’ and ‘Alborea’ situated very close to each other. All the commercial cultivars, except ‘Ortanique’, were located to the right of the MFA space. This distribution indicates that the new cultivars shared physico-chemical and sensory characteristics that were quite different from those displayed by the cultivars currently found on the market. According to the results previously obtained in each harvest independently, the five new cultivars were characterized by attributes such as sour, very aromatic, very intense taste, intense odor when starting to peel, juicy and refreshing taste. Moreover, all the new cultivars showed high acidity and TSS levels, as well as high firmness values.

## 4. Conclusions

Our results indicate that different consumer groups showed distinct preference patterns. Hence, there might not be a unique cultivar that fulfills consumers’ sensory and hedonic expectations. Among the cultivars evaluated at Harvest 1, a large number of consumers showed preference for ‘Clemenules’, while another segment of consumers clearly disliked this variety. Regarding the new cultivars, ‘Alborea’ seemed the most promising variety as it was highly appreciated by most consumers. At Harvest 2, the three new cultivars were preferred to the commercial ones. Among them, ‘Tri-703’ was the most liked by consumers. In fact, this variety obtained the highest liking scores of all the 11 cultivars evaluated in this study.

This is the first time that the sensory profile of mandarin cultivars has been described by consumers. The five new cultivars shared similar sensory profiles, characterized mainly by an intense and refreshing taste, and by being very aromatic and having a juicy texture. These characteristics clearly differentiated the new cultivars from the commercial ones.

Our results corroborate that CATA questions are a valuable tool to describe difference in sensory characteristics among cultivars. Moreover, the relationship between the physico-chemical properties and the sensory profile observed in this study suggests bidirectional data validity. That is, consumer data are valid for characterizing difference among cultivars, while instrumental data may be useful for predicting difference in consumer perceptions among cultivars.

## Figures and Tables

**Table 1:** Physico-chemical parameters of the eleven mandarin cultivars.

Harvest	Cultivars	Color (1000a/Lb)	Firmness (%def)	TSS ^1^ (%)	Acidity (g Citric Acid/100 mL)	MI ^2^ TSS/TA)	JY ^3^ (% juice)	Weight (g)
**1**	Alborea	27.7 d	4.1 a	13.4 b	1.02 c	13.08 b	51.6 a	107.4 c
	Coral	20.4 b	4.3 ab	12.6 a	1.10 c	11.50 ab	58.0 b	110.0 c
	Nova	27.8 d	4.7 b	12.4a	0.74 b	16.79 c	57.7 b	95.54 b
	Clemenules	17.0 a	6.3 c	12.1 a	0.44 a	27.60 d	49.2 a	96.0 b
	Tango	24.9 c	7.3 d	11.9 a	1.10 c	10.79 a	51.9 a	81.8 a
**2**	Tri-703	22.2 c	3.3 c	14.5 c	1.25 c	11.6 c	48.7 ab	119.8 a
	Omet	23.5 c	2.1 a	14.6 c	1.55 d	9.45 b	52.0 b	126.1 a
	Matiz	18.6 b	4.3 d	13.4 b	1.46 d	9.19 ab	49.0 ab	107.2 a
	Orri	14.2 a	3.2 c	13.0 b	0.67 a	19.48 d	50.7 b	122.4 a
	Nadorcott	17.8 b	5.4 e	12.0 a	1.07 b	11.29 c	45.8 a	98.6 a
	Ortanique	19.1 b	2.6 b	11.6 a	1.50 d	7.86 a	51.6 b	180.5 b

^1^ TSS, total soluble solids; ^2^ MI, maturity index; ^3^ JY, juice yield. In each harvest, different letters in the same column indicate significant difference among cultivars according to LSD test (*p* ≤ 0.05).

## Supplementary Materials

The following are available online at https://www.mdpi.com/2304-8158/9/4/468/s1, Table S1: Results of the Cochran’s Q test applied to CATA questions for Harvest 1; Table S2: Results of the Cochran’s Q test applied to CATA questions for Harvest 2. Figure S1: Dendrograms obtained from HCA applied to acceptance scores dataset from Harvests 1 (A) and 2 (B); Figure S2: Penalty analysis applied to the dataset of each of the identified clusters 1; Figure S3: Penalty analysis applied to the dataset of each of the identified clusters.
