# Supplementary material for: Consumer Description by Check-All-That-Apply Questions (CATA) of the Sensory Profiles of Commercial and New Mandarins. Identification of Preference Patterns and Drivers of Liking
Source: Foods. 2020 Apr 9;9(4):468. doi: 10.3390/foods9040468 (PMC7230890; doi:10.3390/foods9040468)
Supplement: Supplementary file 1 [file foods-09-00468-s001.pdf]

SUPPLEMENTARY MATERIAL

Consumer description by Check-All-That-Apply questions (CATA) of the sensory profiles of commercial and new mandarins. Identification of preference patterns and drivers of liking.

Table S1. Results of the Cochran's Q test applied to CATA questions for Harvest 1. Frequencies with which each term was mentioned for each of the evaluated mandarin cultivars are shown.

**Harvest 1**	**Mandarin cultivar**				
Term	**Alborea**	**Clemenules**	**Coral**	**Nova**	**Tango**
Intense odour when starting to peel***	85	14	82	24	28
Easy to peel***	57	104	28	36	98
Difficult to peel***	51	11	81	76	19
Hard starting peeling***	49	29	38	68	28
Stain hands when peeling***	75	2	67	33	21
Tasteless/dull***	6	54	10	38	21
Not very sweet**	33	37	44	31	52
Sweet*	63	39	49	46	43
Very sweet***	13	35	4	35	10
Overripe taste***	4	17	9	20	8
Not traditional/novel taste***	22	11	35	16	37
Refreshing taste***	42	17	35	38	34
Traditional mandarin taste**	47	35	25	40	24
Not very sour***	18	89	18	68	32
Sour***	65	14	52	30	59
Very sour***	27	1	38	3	26
Not very aromatic***	10	65	10	55	45
Very aromatic***	68	11	63	22	26
Very intense taste***	35	4	32	16	24
Soft**	7	21	14	22	21
Fibrous***	31	49	36	14	28
Juiceless***	10	29	4	3	12
Juicy***	77	46	75	79	69

Asterisks indicate significant differences at *** $p \leq 0.001$; **$p \leq 0.01$; * $p \leq 0.05$

Table S2. Results of the Cochran's Q test applied to CATA questions for Harvest 2. Frequencies with which each term was mentioned for each of the evaluated mandarin cultivars are shown.

**Harvest 2**	**Mandarin cultivar**					
Term	**Matiz**	**Nadorcott**	**Omet**	**Orri**	**Ortanique**	**Tri-703**
Intense odour when starting to peel***	83	23	52	23	34	52
Easy to peel***	100	119	24	79	4	42
Difficult to peel***	23	8	96	45	132	76
Hard starting peeling***	37	19	101	54	108	55
Stain hands when peeling***	43	17	68	32	90	32
Tasteless/dull***	8	49	4	38	25	5
Not very sweet***	34	47	34	30	64	19
Sweet***	68	49	60	50	32	68
Very sweet***	19	19	20	47	8	36
Overripe taste***	2	17	4	20	14	4
Not traditional/novel taste***	35	31	12	29	17	10
Refreshing taste**	57	39	52	48	40	63
Traditional mandarin taste***	38	23	42	36	21	66
Not very sour***	25	64	16	96	26	44
Sour***	77	50	76	24	43	66
Very sour***	27	4	38	2	53	9
Not very aromatic***	14	58	25	54	39	21
Very aromatic***	82	23	58	21	35	47
Very intense taste***	35	10	50	16	28	37
Soft***	11	43	7	11	22	10
Fibrous (n.s)	31	45	52	42	37	40
Juiceless**	5	13	7	19	6	8
Juicy**	85	62	87	76	71	86

Asterisks indicate significant differences at *** $p \leq 0.001$; **$p \leq 0.01$; * $p \leq 0.05$; (n.s) indicates no significant difference ($p \leq 0.05$)

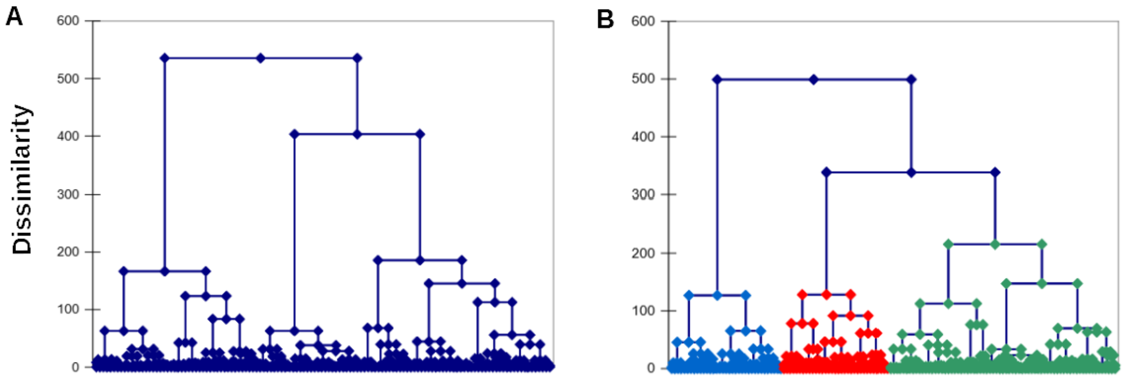

Figure S1. Dendrograms obtained from HCA applied to acceptance scores dataset from Harvest 1 (A) and Harvest 2 (B).

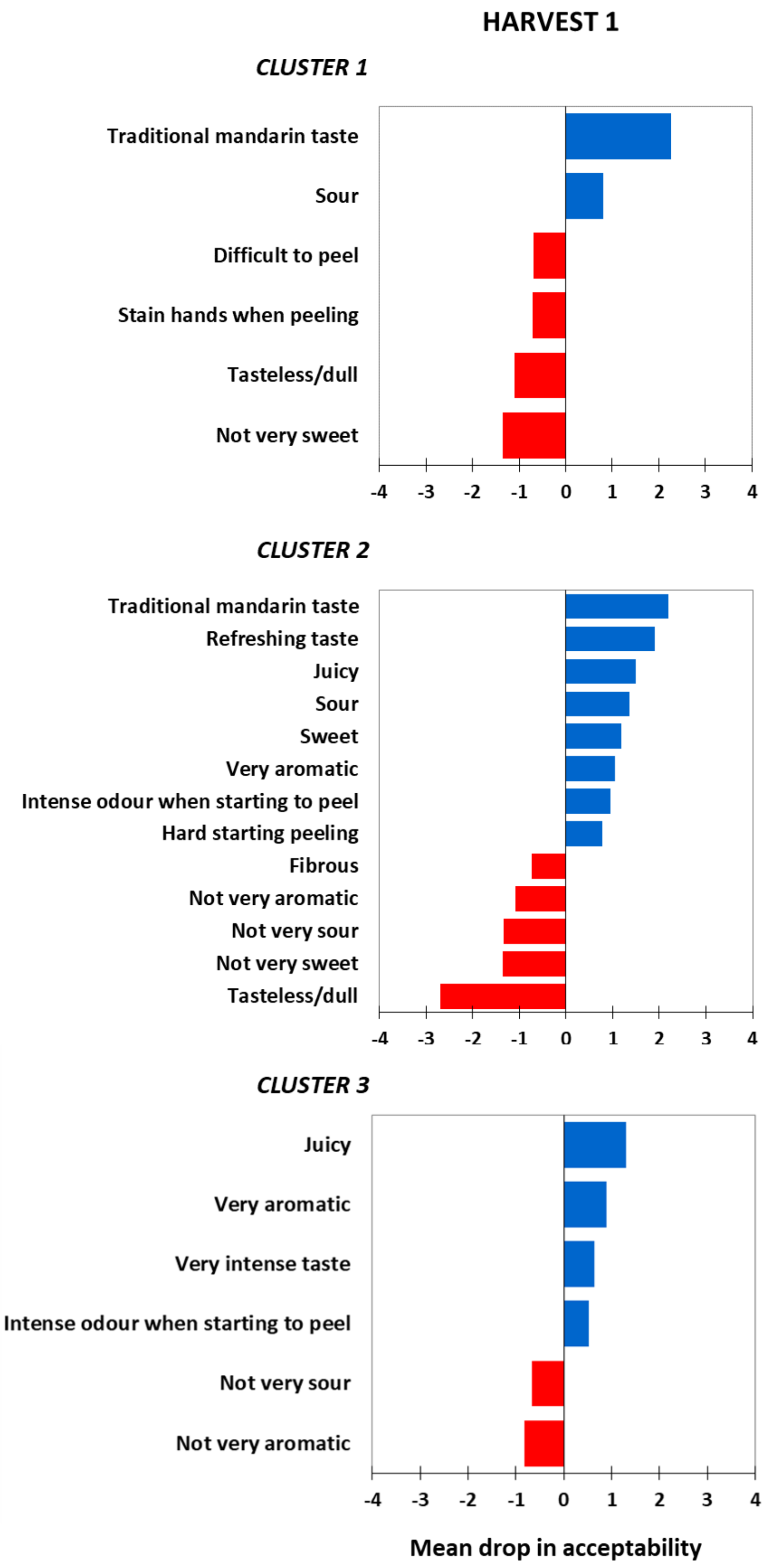

Figure S2. Penalty analysis applied to the dataset of each of the identified clusters. Variation in liking across cultivars and consumers in relation to attributes (positive in blue and negative in red). Only attributes with a significant effect ($p < 0.05$) and an occurrence higher than 20 % of cases were included.

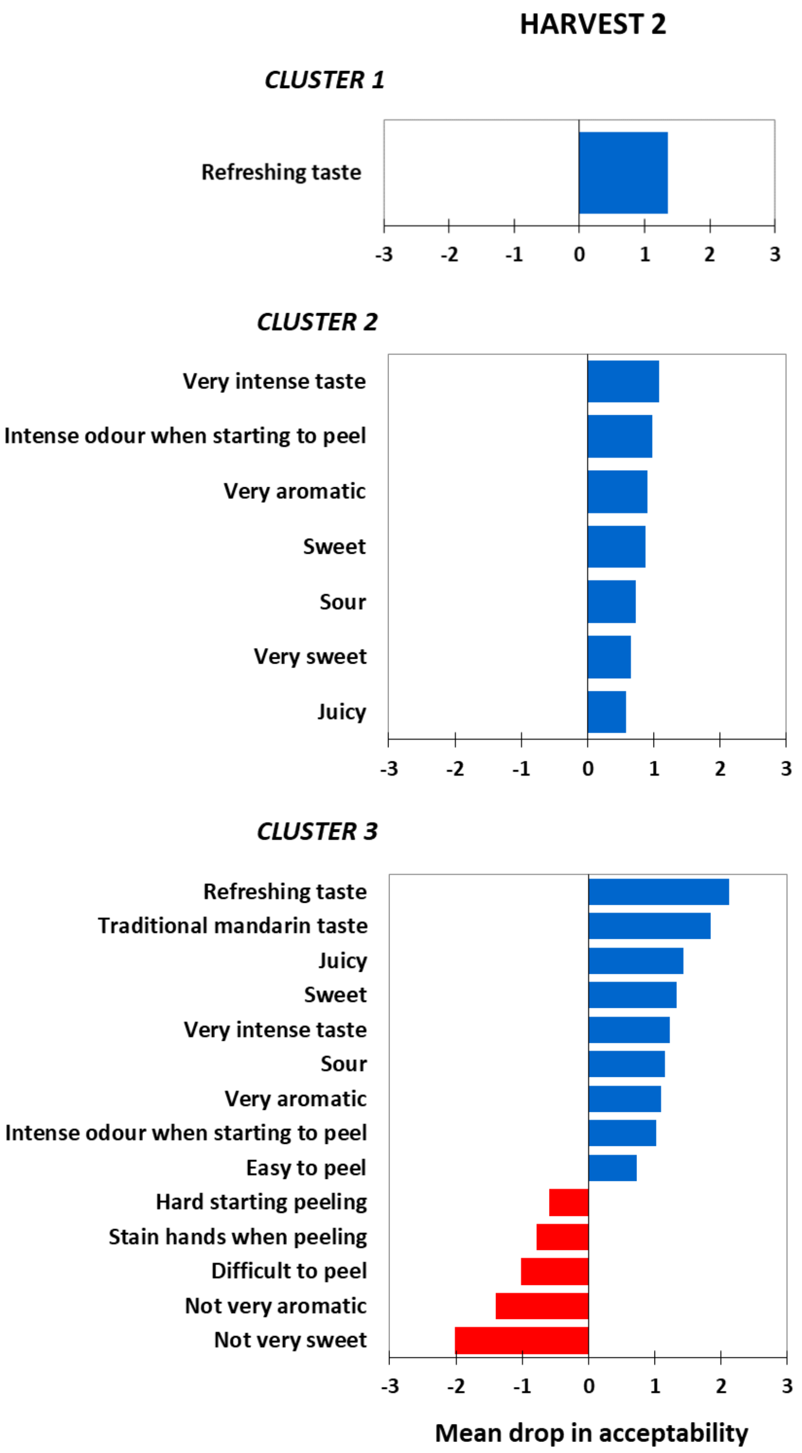

Figure S3. Penalty analysis applied to the dataset of each of the identified clusters. Variation in liking across cultivars and consumers in relation to attributes (positive in blue and negative in red). Only attributes with a significant effect ($p < 0.05$) and an occurrence higher than 20% of cases were included.
